# A global perspective on the abundance, diversity and mobility of antibiotic resistance genes in *Escherichia coli*

**DOI:** 10.3389/fvets.2024.1442159

**Published:** 2024-11-13

**Authors:** Yun Qing, Zhongai Zou, Guolian Jiang, Lingshi Qin, Kehui Liu, Zongbao Liu

**Affiliations:** ^1^Key Laboratory of Ecology of Rare and Endangered Species and Environmental Protection (Guangxi Normal University), Ministry of Education, Guilin, Guangxi, China; ^2^Guangxi Key Laboratory of Landscape Resources Conservation and Sustainable Utilization in Lijiang River Basin, Guangxi Normal University, Guilin, Guangxi, China; ^3^College of Life Sciences, Guangxi Normal University, Guilin, Guangxi, China; ^4^College of Environment and Public Health, Xiamen Huaxia University, Xiamen, Fujian, China

**Keywords:** *Escherichia coli*, antibiotic resistance genes, mobile genetic elements, gene transfer, global perspective

## Abstract

**Introduction:**

*Escherichia coli* (*E. coli*), a ubiquitous opportunistic pathogen, poses a growing threat to human health due to the increasing prevalence of antibiotic resistance. However, a comprehensive understanding of the global distribution, diversity, and transmission of antibiotic resistance genes (ARGs) in *E. coli* remains lacking, hindering effective strategies to combat resistance.

**Methods:**

In this study, we analyzed 94,762 *E. coli* genome sequences obtained from the NCBI database using advanced bioinformatics tools. ARGs were identified by comparing sequences against a custom ARG database using BLAST. Mobile genetic element (MGE)-associated ARGs were identified by matching with ISfinder databases. Global distribution of ARGs was analyzed by clustering mobile ARG sequences with 99% genetic similarity.

**Results:**

Our analysis revealed that 50.51% of the *E. coli* genome sequences contained ARGs, totaling 301,317 identified ARG sequences. These ARGs were categorized into 12 major classes and 229 subtypes. Notably, ARGs associated with multi-drug resistance (MDR), *β*-lactams, macrolide-lincosamide-streptogramins (MLS), tetracyclines, and aminoglycosides were particularly abundant, with the subtypes *mdtK*, *macB*, and *ampC* being especially prevalent. Additionally, significant differences in ARG abundance and diversity were observed across countries, with higher diversity found in high-income nations. Furthermore, 9.28% of the ARG sequences were linked to MGEs, accounting for 98.25% of all ARG subtypes. Notably, 4.20% of mobile ARGs were identified in over 20 countries, with *β*-lactam and aminoglycoside ARGs being the most widespread.

**Discussion:**

This study provides a comprehensive overview of the global distribution and transmission of ARGs in *E. coli*. The high abundance of MDR and *β*-lactam-related ARGs, along with their widespread transmission across countries, highlights the urgent need for global surveillance and control measures. Furthermore, the strong association between ARGs and MGEs underscores the role of horizontal gene transfer in the spread of resistance. The observed variations in ARG diversity between countries suggest that socioeconomic factors, such as healthcare infrastructure and antibiotic usage patterns, significantly influence ARG prevalence. These findings are crucial for informing global strategies to mitigate the spread of antibiotic resistance and improve public health outcomes.

## 1 Introduction

*Escherichia coli* (*E. coli*), a Gram-negative bacterium ubiquitously present in the intestines of humans and animals, is a common opportunistic pathogen encountered in clinical settings (1). The widespread application of antibiotics has exacerbated the issue of antibiotic resistance in *E. coli*, broadening its spectrum and posing a significant challenge to global public health (2). Currently, *E. coli* has demonstrated varying degrees of resistance to antibiotics such as *β*-lactams, aminoglycosides, tetracyclines, and fluoroquinolones (3–6). The genesis of resistance is primarily attributed to the presence and dissemination of specific antibiotic resistance genes (ARGs), such as the *bla*_CTX-M_ genes, which is one of the key genes in conferring resistance to *β*-lactam antibiotics in *E. coli* (7). Notably, these ARGs are not a byproduct of modern human activities but have existed in nature before the discovery of antibiotics (8, 9). In fact, human activities have facilitated the selection and transfer of these genes from environmental and host cellular origins to various bacteria, leading to widespread antibiotic resistance (10, 11).

Mobile genetic elements (MGEs), such as plasmids, integrons (Int), transposons, and insertion sequences (IS), play a crucial role in the propagation of ARGs by promoting horizontal gene transfer (HGT) among different bacteria (12). The presence of MGEs significantly enhances the mobility of ARGs, and studies have shown a positive correlation between the abundance of MGEs and ARGs (13–16). Consequently, mobile ARGs associated with MGEs pose a substantial threat to human health, necessitating widespread attention from both the public and the scientific community.

While numerous studies have reported on the antibiotic resistance of *E. coli* and its ARGs, they often focus on specific types of ARGs, environments, or geographical areas, or are based solely on strains isolated from human clinical infections. This limited scope of research fails to capture the full spectrum of ARGs related to *E. coli* resistance, leading to a lack of comprehensive understanding of its global distribution and transmission patterns. Furthermore, the dynamics of the transmission of ARGs, particularly the role of MGEs in facilitating the cross-border and cross-species dissemination of resistance, requires further elucidation. Therefore, research on the global distribution and transmission of *E. coli* ARGs is crucial for understanding its resistance mechanisms, developing effective control strategies, and guiding national and global public health priorities and treatment decisions.

In light of these challenges, the present study harnesses an extensive dataset comprising 94,762 *E. coli* genome sequences from the National Center for Biotechnology Information (NCBI) database encompassing 104 countries and regions, undertaking a thorough bioinformatics analysis to map the global prevalence characteristics of ARGs within *E. coli* The investigation includes an analysis of the variance in ARGs composition across different nations, the dynamics of interaction between ARGs and MGEs, and their prospective transmission risks. The findings will provide a scientific basis for formulating strategies to control the spread of antibiotic resistance in *E. coli* and other pathogenic bacteria worldwide.

## 2 Materials and methods

### 2.1 Data collection

This study accessed the NCBI Reference Sequence (RefSeq) database^1^ to retrieve *E. coli* genome sequences available up to April 16, 2023, from 104 countries and regions using “*Escherichia Coli*” as the search keyword. Sequences were further filtered by setting minimum length criteria, specifically sequences ≥300,000 bp for chromosomes and ≥ 2,000 bp for plasmids. Both GenBank and FASTA format files of the sequences were downloaded to obtain detailed genomic information and nucleotide sequences, respectively. Python scripts were employed to convert the downloaded GenBank files (sequence.gb) into CSV format, enabling the systematic compilation of data regarding the isolation sources (human, animal, food, and environmental), country of origin, collection date, and genetic background (plasmid or chromosomal).

### 2.2 Identification of ARGs

The Prodigal v2.6.3 software (17) was employed to predict functional genes within the downloaded *E. coli* genome sequences, preserving both the predicted genes and amino acid sequences. These sequences were then compared against a laboratory-constructed ARGs database developed in our previous study (18), integrating known ARGs from databases such as CARD (Comprehensive Antibiotic Resistance Database), ARDB (Antibiotic Resistance Genes Database), CGE (Center for Genomic Epidemiology), and NCBI nr database, after removing duplicates and erroneous sequences, and categorizing the ARGs. BLAST 2.13.0 software (19) facilitated this comparison, with sequences exhibiting ≥80% identity and ≥ 70% coverage classified as ARGs (20, 21).

### 2.3 Abundance of ARGs

ARGs abundance was calculated as “copies of ARG per million base pairs (bp)” (CPM), using the following formula (21):


$$\begin{array}{l} \mathrm{Abundance}\;\left(CPM\right)=\\ \overset{n}{\underset{1}{\sum }}\frac{N_{ARG}\times L_{\mathrm{identified}\;ARG\;\mathrm{sequence}}/L_{ARG\;\mathrm{reference sequence}}}{\mathrm{Total genomic length}}\times 1000000 \end{array}$$

where N_ARG_ represents the count of identified ARGs sequences; L_identified ARG sequence_ denotes the length of sequences identified as ARGs; L_ARG reference sequence_ refers to the length of ARGs reference sequences in the database; and Total genomic length is the total length of the *E. coli* genome sequences downloaded.

### 2.4 Identification of MGE-associated ARGs

Genome sequences of *E. coli* carrying ARGs were extracted utilizing a custom Perl script. Plasmid genome sequences were identified based on the genetic background information contained within the GenBank files. These ARG-carrying genomes were then compared against the INTEGRALL (22) and ISfinder (23) databases using BLAST, identifying Int and IS sequences by selecting sequences with ≥80% identity and ≥ 70% coverage (21). Int-related ARGs were identified by examining genes upstream and downstream of the Int, while ARGs sequences within 3 genes or 5,000 bp of IS sequences were deemed IS-related (24). Cumulatively, ARGs situated on plasmids or linked to Int and IS sequences were designated as MGE-related ARGs.

### 2.5 Global distribution of MGE-associated ARGs

A self-written Perl script was used to extract MGE-related ARG sequences, which were further processed with USEARCH (v10.0.240) software to eliminate duplicate sequences, forming a preliminary database of mobile ARGs in *E. coli*. Using BLAST, ARGs sequences from different countries were compared against this database to reveal the global distribution of mobile ARGs sequences or highly homologous sequences, setting a 99% genetic homology threshold. Similarly, the same methodology was applied to analyze the same or highly homologous ARG sequences in *E. coli* from different sources.

### 2.6 Data analysis

Statistical analyses and bar graph creations were performed using Microsoft Excel 2016. Heatmap analyses and creations were conducted using the pheatmap package in R v3.6.3.^2^ Bubble charts of *E. coli* ARGs abundance by phenotypic class were generated using the OmicShare platform.^3^ Venn diagrams of ARGs and MGE associations and Rarefaction curves for ARGs from different countries were plotted using the Biozeron cloudplatform (25).^4^ Global dissemination maps of mobile ARGs and image adjustments were made using Adobe Illustrator.

## 3 Results

### 3.1 Prevalence analysis of ARGs in *Escherichia coli*

**Figure 1:**
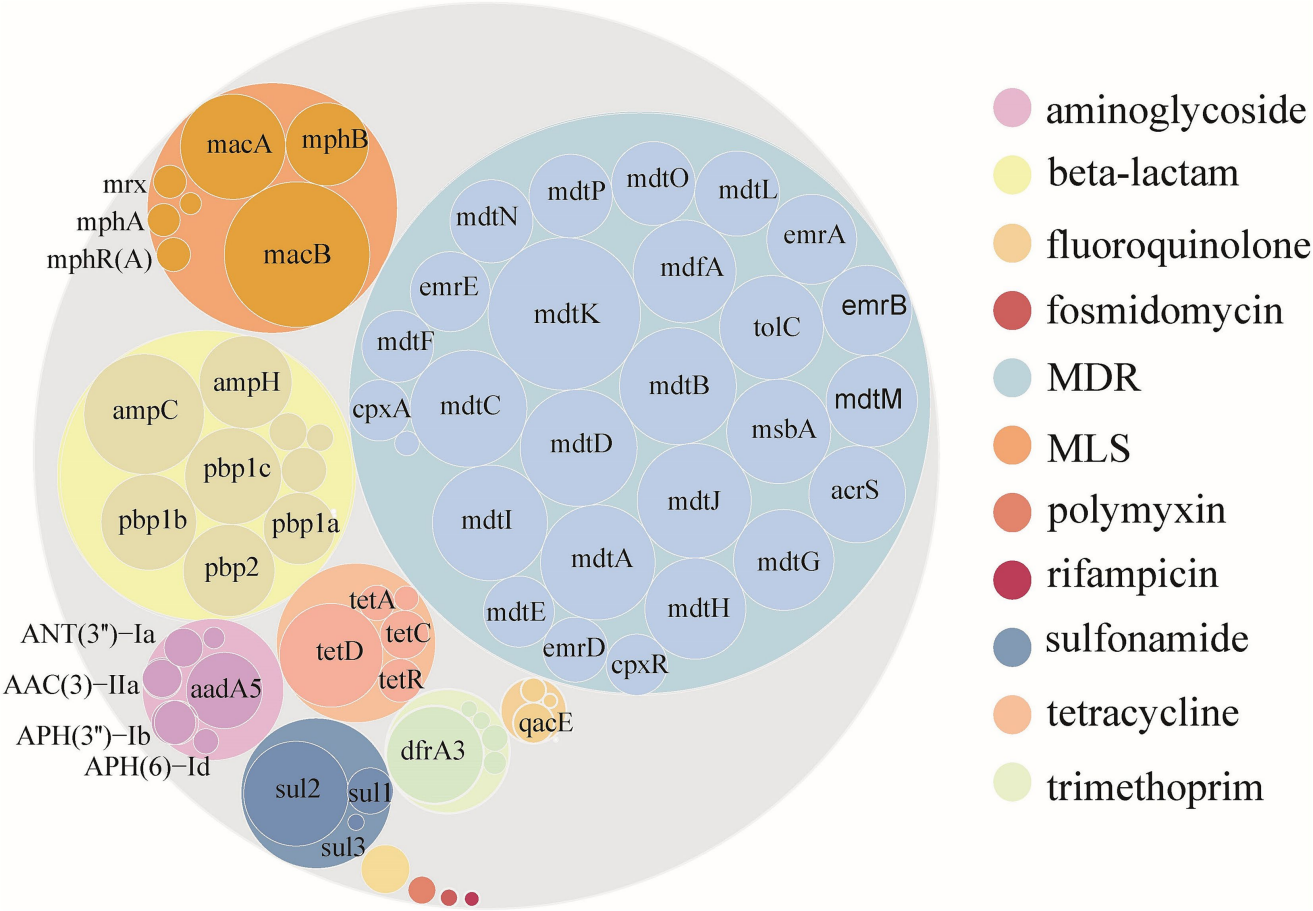
Abundance of ARGs carried by *Escherichia coli* globally. Color according to ARGs type. The outer and inner circles represent the ARGs type and subtype, respectively. The size of the circle is directly proportional to the abundance of ARGs.

As of April 16, 2023, an analysis of 94,762 *E. coli* genomes from the NCBI RefSeq database revealed that 50.51% (47,865) contained ARGs, totaling 301,317 ARGs sequences across 12 major classes and 229 subtype (Supplementary Table S1; Supplementary Figure S1). The distribution of resistance types varied, with multi-drug resistant (MDR) genes being the most abundant, representing 59.67% of the total abundance. Subsequently, genes associated with *β*-lactamases, macrolide-lincosamide-streptogramins (MLS), tetracyclines, aminoglycosides, sulfonamides, and trimethoprim followed, with abundances of 1.36, 0.97, 0.37, 0.32, 0.31, and 0.26 CPM, respectively. ARGs for the other phenotypes together accounted for only 1.61% of the total abundance. The *mdtK* gene subtype emerged as the most prevalent among *E. coli*, with an abundance of 0.51 CPM, succeeded by *macB*, *mdtC*, *mdtB*, *mdtD*, *ampC*, and others, which displayed considerable abundances ranging from 0.48 to 0.31 CPM. Notably, of the top 30 ARGs subtypes by abundance, 19 were MDR, 5 were *β*-lactamases, 3 were MLS, and the remaining 3 corresponded to sulfonamides, tetracyclines, and trimethoprim, respectively (Figure 1).

### 3.2 Diversity and abundance of ARGs in *Escherichia coli* across different countries

**Figure 2:**
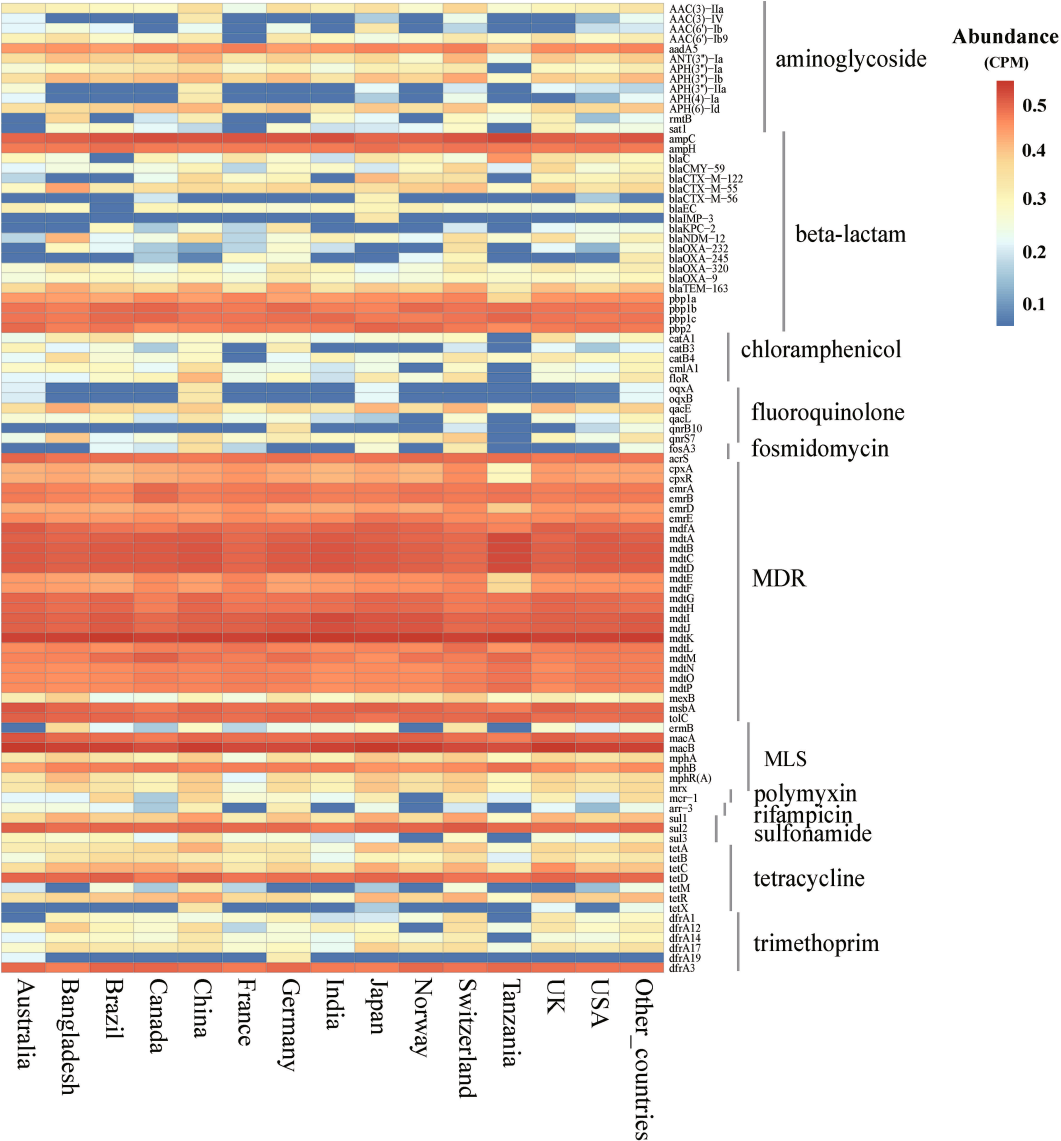
Abundance of ARGs subtypes in *E. coli* among different countries. (abundance >1 × 10^−2^ CPM in at least one country). CPM: copies of ARG per million base pairs.

Using *E. coli* genome samples and their origin data from the NCBI database, this study analyzed the 14 countries with the most genome sequences to assess ARGs abundance and diversity. The analysis revealed significant variability in ARGs composition across these countries (Kruskal-Wallis, *p* < 0.01), identifying 12 major classes and 229 subtypes of ARGs (Supplementary Table S2). Although ARG abundances varied widely among countries, major ARGs types, such as MDR, MLS, *β*-lactamases, and tetracyclines, were consistently prevalent across the board (Supplementary Figure S1). Additionally, 97 ARGs subtypes were found to exceed an abundance of 1 × 10^−2^ CPM in at least one country, with 60 of these subtypes being prevalent across many nations. Notably, subtypes such as *mdtK*, *macB*, *ampC*, and *mdtD* were predominant in most countries (Figure 2).

**Figure 3:**
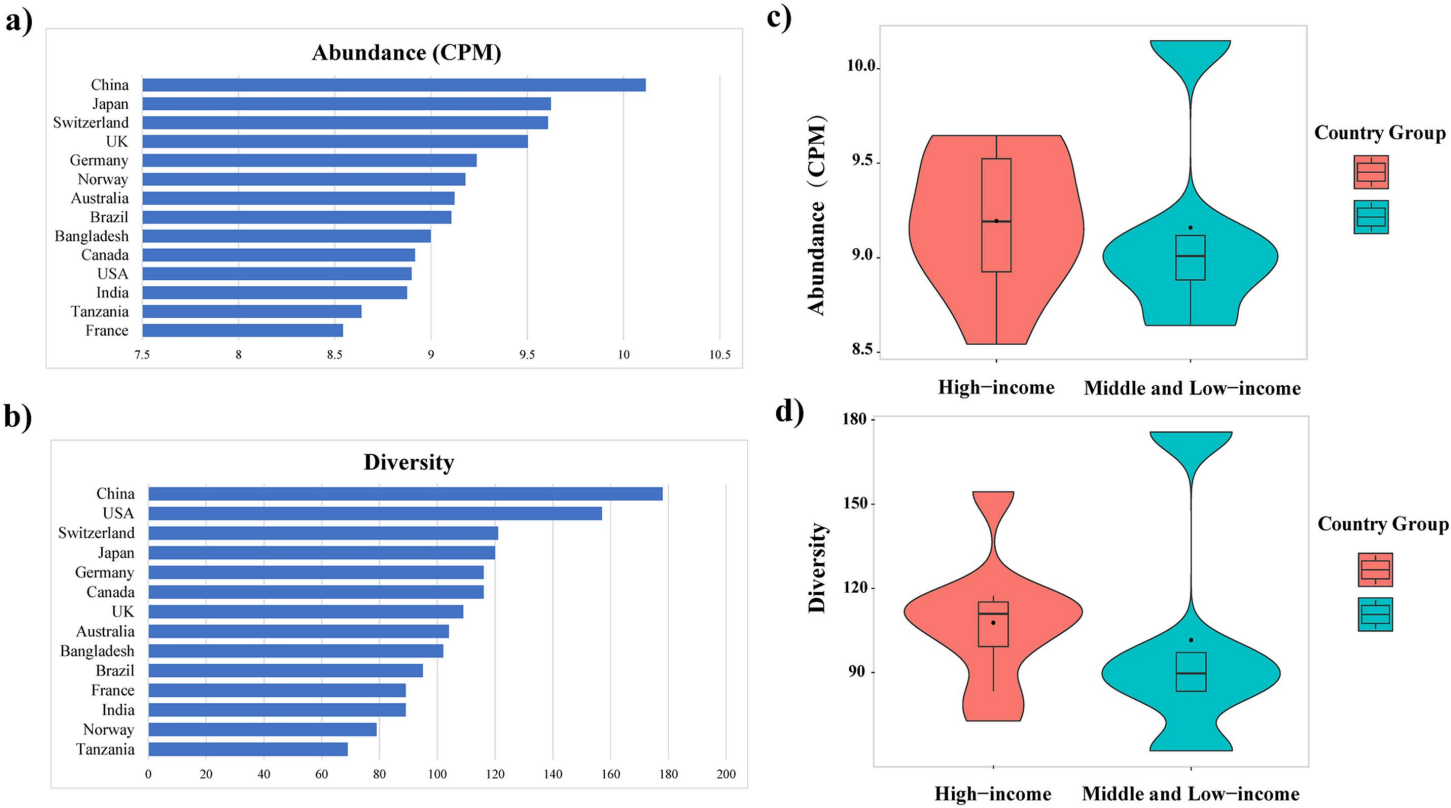
Variation in the abundance (A) and diversity (B) of ARGs across countries; Abundance (C) and diversity (D) of *E. coli* ARGs in high-income and middle and low-income countries.

Among the countries analyzed, the total abundance of ARGs in *E. coli* genomes from China was the highest, exceeding 10 CPM (Figure 3A). Similarly, further analysis of ARG diversity revealed that China exhibited the most diverse ARG profile, with 176 subtypes, followed by the United States, Switzerland, Japan, Canada, and Germany, which had 157, 121, 120, 116, and 116 subtypes, respectively. In contrast, Tanzania displayed the lowest diversity, with only 69 subtypes identified (Figure 3B). With the exception of China, high-income countries demonstrated significantly higher abundance and diversity of *E. coli* ARGs compared to low- and middle-income countries (Welch’s *t*-test, *p* < 0.05) (Figures 3C, 3D). The study also generated rarefaction curves for ARGs (Supplementary Figure S2), showing a plateau in the number of detectable ARGs subtypes as sequence numbers increased, indicating sufficient sample sizes for the analysis. Additionally, a time series analysis from 2010 to 2016 indicated an upward trend in ARGs abundance in *E. coli* in China, which, after a decline in 2017, peaked in 2019 and began to decrease annually from 2020 onwards (Supplementary Figure S3).

### 3.3 Distribution of ARGs in *Escherichia coli* across different sources

The diversity of ARGs in *E. coli* from various sources—human, animal, food, and environmental—was assessed. Human-derived genomes exhibited the highest diversity, containing 194 distinct ARG subtypes, followed by environmental genomes with 152 subtypes, animal-derived genomes with 141 subtypes, and food sources with the lowest diversity at 110 subtypes (Supplementary Table S3; Supplementary Figure S4b). In terms of abundance, environmental genomes demonstrated the highest abundance of ARGs at 9.67 CPM, closely followed by animal-derived genomes at 9.47 CPM. Human-derived genomes showed a slightly reduced abundance of 9.46 CPM, while food-derived *E. coli* exhibited the lowest abundance at 8.90 CPM (Supplementary Table S3; Supplementary Figure S4a).

Supplementary Figure S5 illustrates the abundance of different ARG types in *E. coli* from these sources. Among the ARG classes analyzed, MDR genes exhibited the highest abundance across all sources, with values ranging from 5.40 CPM in food-derived genomes to 5.77 CPM in animal-derived genomes. Supplementary Figure S6 further demonstrates the detailed distribution and abundance of ARG subtypes in *E. coli* from different sources. The MDR genes *mdtK*, *mdtC*, *mdtB*, *mdtD*, *mdtA*, *mdtI*, *mdtJ*, *macA*, the MLS resistance gene *macB*, the beta-lactam resistance gene *ampC*, the sulfonamide resistance gene *sul2*, and the tetracycline resistance gene *tetD* exhibited high abundance across various sources. Overall, there were notable variations in both the number of distinct ARG subtypes and their corresponding abundance among the different origins.

### 3.4 MGE-related ARGs

**Figure 4:**
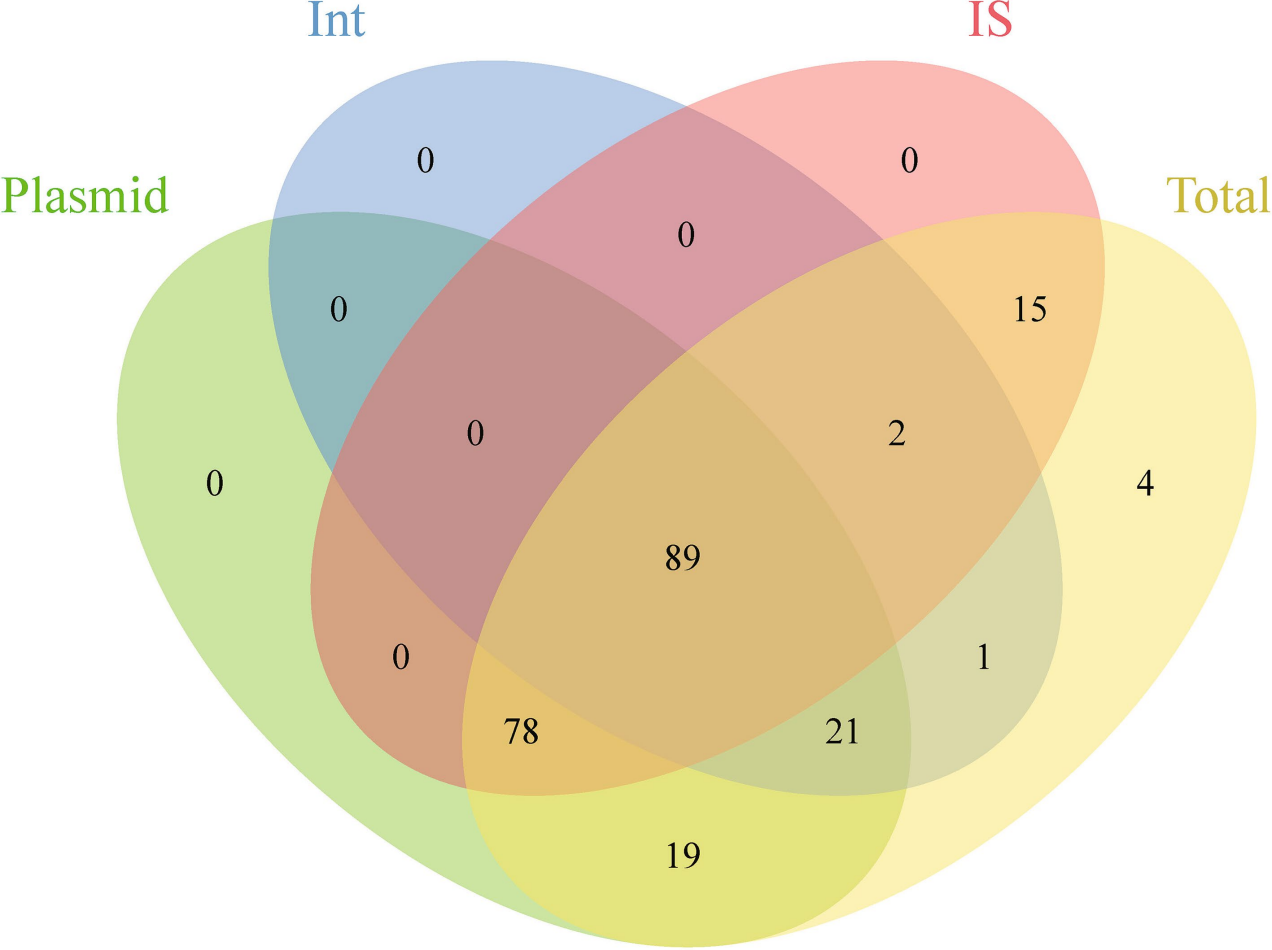
Venn diagram showing the shared and unique ARGs subtypes across different genetic backgrounds.

This study evaluated the potential for ARGs dissemination in *E. coli* by analyzing ARGs linked to three principal MGEs: plasmids, Int, and IS (Supplementary Table S4). The investigation identified 22,940 plasmid sequences, 3,194 Int, and 424,732 IS within the *E. coli* genomes examined. Notably, *IS3* and *IS1* elements constituted 44.75% of all IS elements, while *Int1* accounted for 96.30% of all Int elements. Analysis showed that approximately 9.78% of ARGs (29,475/301,317) were associated with these MGEs, spanning 12 ARGs categories. Among these, 96.54% of Int-related ARGs were linked to *Int1*; for Int, 33.60% related to *IS6*. ARGs associated with IS demonstrated significant abundance and were highly linked to MGEs. Additionally, 98.25% of the 229 analyzed ARGs subtypes were connected to MGEs, with 89 subtypes associated with all three MGE types, predominantly including aminoglycosides, *β*-lactamases, fluoroquinolones, MLS, and trimethoprim. Furthermore, 101 subtypes were related to two MGE types, with 78 linked to both plasmids and Int, mainly encompassing β-lactamases and MDR genes (Figure 4; Supplementary Table S5).

### 3.5 Global distribution of mobile ARGs

**Figure 5:**
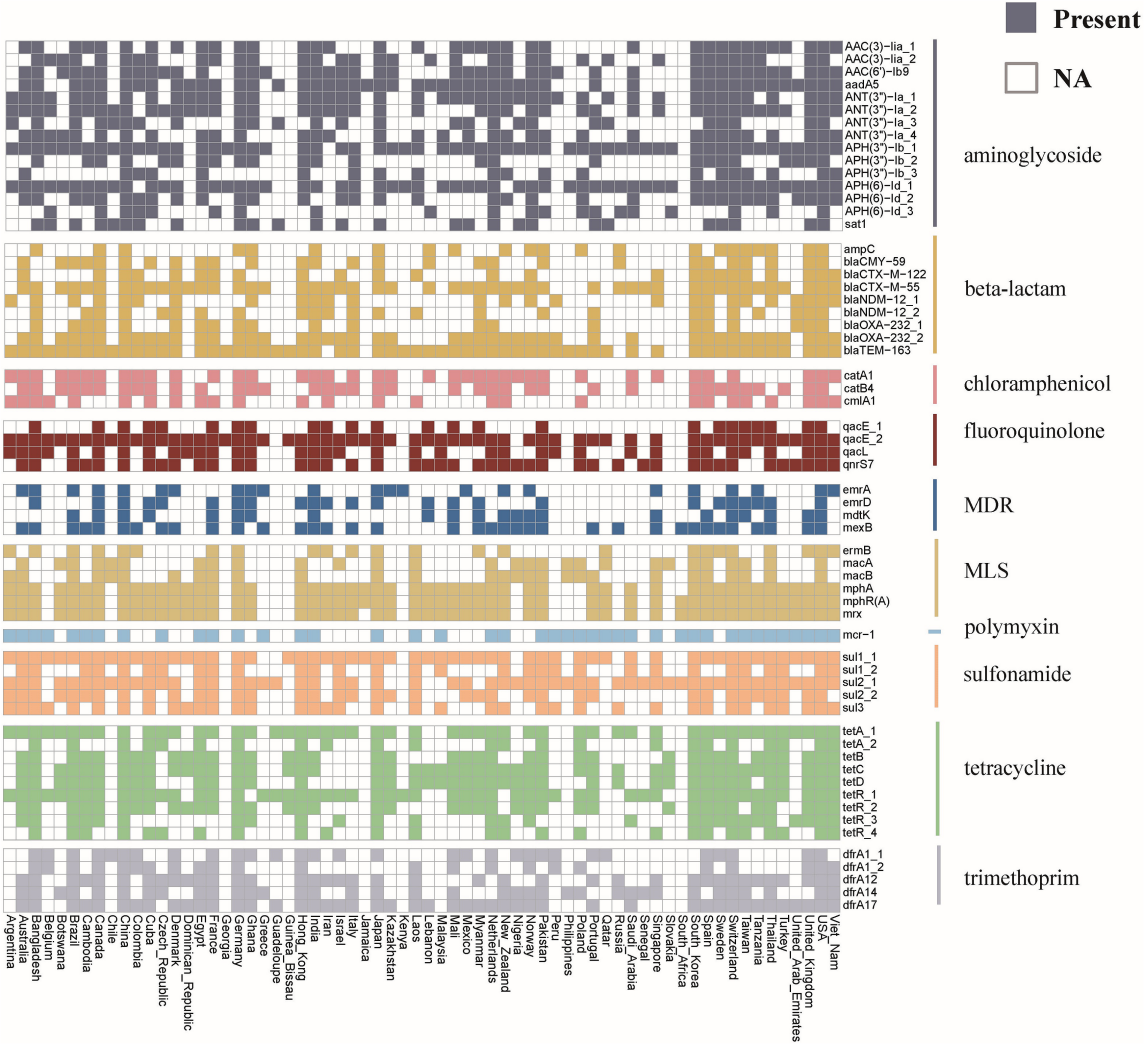
Global distribution of mobile ARGs in *E. coli*. Different colors represent different categories of ARGs. The colored blocks indicate the presence of ARG in the corresponding country, while the white blocks indicate its absence.

**Figure 6:**
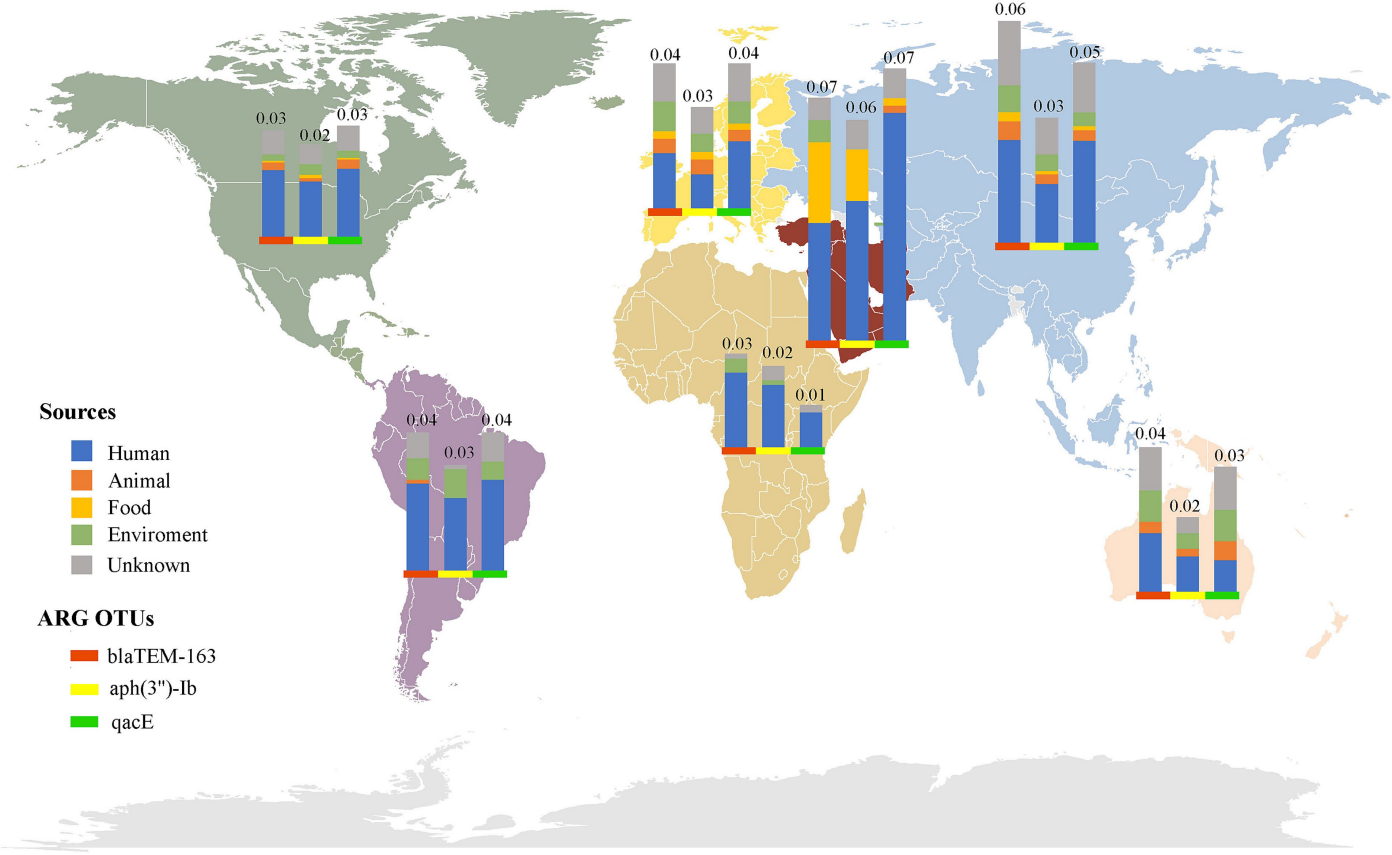
Global distribution of the top three abundant mobile ARGs OTUs in *E. coli*. The bars of different colors represent different ARG OTUs, and the height of the bars represents the abundance (CPM) of the ARGs OTUs in the local area.

Investigating the worldwide distribution of mobile ARGs, this study performed a cluster analysis based on 99% homology, identifying 1,451 ARGs operational taxonomic units (OTUs; Supplementary Table S6). The findings revealed that 4.14% (60 OTUs) of mobile ARGs were present in over 20 countries globally (Figure 5). Significant numbers of mobile ARGs were identified in China, United States, Canada, Switzerland, Japan, Germany, South Korea, and Thailand among the 104 countries and regions surveyed. Notably, genes such as *β*-lactamase *bla*_TEM-163_, aminoglycosides *aph(3″)-Ib* and *aph(6)-Id*, sulfonamides *sul1* and *sul2*, tetracyclines *tetR* and *tetA*, and MLS *mphR(A)*, *mphA*, *mrx* genes were prevalent in over 50 countries. Detailed analysis of the distribution of three widely dispersed ARGs—*bla*_TEM-163_ (Accession No. NZ_CP063490.1), *aph(3″)-Ib_1* (Accession No.: NZ_RRDM01000134.1), and *qacE_2* (Accession No. NZ_JABACN010000007.1)—showed their presence in at least one of 71 countries as of April 2023. These ARGs were notably widespread in China, the United States, and Japan, spreading to 63, 58, and 58 countries, respectively. Analysis of the continental abundance changes for these ARGs indicated their extensive distribution across all continents except Antarctica, with Asia, Europe and North America as primary regions (Figure 6; Supplementary Table S7). These globally distributed ARGs predominantly originate from humans, followed by environmental sources. Further analysis revealed that *E. coli* genomes from four different origins (human, animal, food, and environmental) shared a total of 146 (146/1760) identical or highly homologous ARGs (Supplementary Figure S7; Supplementary Table S8). Similarly, resistance genes such as *bla*_TEM-163_, *qacE*, *sul1*, *mphR(A)*, *mphA*, *mrx*, *sul2*, *tetA*, *dfrA17*, and *bla*_CTX-M-55_ were widely distributed across various sources (Supplementary Table S8).

## 4 Discussion

*E. coli* possesses a remarkable ability to adapt to a range of environmental conditions, facilitating the rapid acquisition of antibiotic resistance (26). This analysis of 94,762 *E. coli* genomes found that 50.51% harbored ARGs, highlighting both the significant diversity and abundance of these genes. The ubiquitous presence of ARGs emphasizes the global challenge posed by the misuse and overuse of antibiotics, accelerating the proliferation of resistance (27). Notably, the prevalence of MDR genes, which constitute approximately 60% of all detected ARGs, highlights the robustness of *E. coli* against multiple antibiotics, underscoring the ineffectiveness of monotherapy in treating certain infections (28). Correspondingly, Zhang et al. (29) have observed a similar trend in the abundance of MDR genes in clinical isolates, indicating that these genes provide a significant survival benefit under diverse antibiotic pressures. This necessitates a reevaluation of current healthcare strategies and policy frameworks. The widespread prevalence of ARGs associated with *β*-lactamases, MLS, tetracyclines, aminoglycosides, sulfonamides, and trimethoprim indicates the global use of these antibiotic classes and highlights the consequent challenges of resistance. The marked prominence of the *mdtK* gene subtype observed in *E. coli* genomes warrants special attention. Its prevalence, alongside other subtypes such as *macB* and *ampC*, is consistent with the observations made by Zhang et al. (30), who reported an increasing trend in the acquisition of these subtypes, especially in hospital environments characterized by intensive antibiotic usage.

Investigating *E. coli* genomes across nations revealed distinct geographic patterns in ARGs diversity, shedding light on regional resistance traits. For instance, certain ARGs prevalent in one country may be scarce in another, reflecting variations in antibiotic use, hygiene practices, and infection control measures. A significant aspect of our findings was the variation in ARGs diversity between high-income and middle- and lower-income countries. Except for China, high-income countries exhibited higher ARGs diversity, which could be attributed to more robust genomic surveillance and diverse antibiotic usage patterns (31). In contrast, the low diversity observed in countries like Tanzania, which had only 69 identified subtypes, could reflect limited access to diverse antibiotics and fewer resources for genomic surveillance (32, 33). While the abundance of ARGs exhibited significant variability across countries, certain types such as MDR, MLS, *β*-lactamases, and tetracyclines were consistently prevalent worldwide. This ubiquity highlights the successful adaptation of these resistance mechanisms within *E. coli* populations and various ecological environments. Notably, the subtypes *mdtK*, *macB*, *ampC*, and *mdtD* are predominantly present in most countries, associated with resistance to critically important antibiotics. This poses substantial challenges to global treatment protocols and infection control strategies. For example, the *ampC* β-lactamase gene confers resistance against a wide range of β-lactam antibiotics, complicating the management of prevalent bacterial infections. Supporting these findings, recent research indicates a similar pattern of ARGs distribution globally. A large number of studies have found that ARGs such as *ampC* were prevalent not only in clinical isolates (34) but also increasingly in environmental samples (35, 36), indicating a widespread dissemination beyond healthcare facilities. Additionally, Li et al. (37) documented a rise in the prevalence of the *mdtK* gene subtypes, particularly in hospital environments characterized by intensive use of antibiotics.

China exhibited the highest levels of ARGs diversity and abundance in *E. coli* among the countries surveyed. This prominence is closely linked to China’s role as the world’s largest producer and consumer of antibiotics, profoundly influencing the epidemiology of antibiotic resistance (38, 39). Notably, fluctuations in ARGs abundance over the past decade in China depict a complex pattern of resistance evolution. A time series analysis from 2010 to 2019 demonstrated a rising trend in ARGs abundance, reaching its apex in 2019, attributable to widespread antibiotic use in both clinical and agricultural sectors (40). However, a decline was observed beginning in 2020, coinciding with the onset of the COVID-19 pandemic. During this period, significant changes in healthcare practices, particularly in the management of bacterial infections suspected of being secondary to viral infections, likely impacted antibiotic prescribing practices. Comparatively, other regions globally have reported diverse impacts of the COVID-19 pandemic on antibiotic resistance. For instance, some underdeveloped African countries experienced initial increases in ARGs, possibly due to heightened antibiotic usage during the early stages of the pandemic (41). Regulatory measures have since curbed ARGs proliferation, highlighting the link between antibiotic use and resistance management, as well as the vital role of policy in public health and ecological integrity (42).

The assessment of ARG diversity and abundance in *E. coli* from various sources reveals critical insights into the ecology of resistance genes. The observation that human-derived genomes exhibit the highest diversity, with 194 distinct ARG subtypes, underscores the significant role of human activities and antibiotic use in shaping resistance profiles. Environmental genomes, with 152 distinct ARG subtypes, highlight the importance of environmental reservoirs in harboring resistance genes. These environments can serve as reservoirs and vectors for ARGs, facilitated by the selective pressure from anthropogenic factors, including agricultural runoff and waste disposal. The high abundance of ARGs in environmental genomes (9.67 CPM) further emphasizes the critical role these sources play in the dissemination of resistance. The relatively lower diversity of ARGs in animal-derived (141 subtypes) and food-derived genomes (110 subtypes) suggests that while these sources contribute to the resistance landscape, they may do so in a more constrained manner. The high abundance of ARGs in animal-derived *E. coli* (9.47 CPM) could be attributed to the extensive use of antibiotics in livestock, which creates selective pressure that promotes the survival and proliferation of resistant strains.

The shared presence of 146 identical or highly homologous ARGs across *E. coli* genomes from human, animal, food, and environmental sources suggests a dynamic exchange of genetic material among these reservoirs. This finding highlights the potential for horizontal gene transfer, which can occur in diverse environments, thereby facilitating the rapid dissemination of resistance traits (43). The overlap of ARGs across different sources raises critical questions regarding the pathways through which resistance genes may be transmitted, particularly from human environments to environmental and food sources. The widespread distribution of specific resistance genes, such as *bla*_TEM-163_, *qacE*, and *sul1* (Supplementary Table S8), underscores the interconnectedness of these reservoirs. This interconnectedness necessitates a One Health approach to effectively monitor and manage antibiotic resistance, incorporating strategies that encompass human health, veterinary practices, and environmental management.

MGEs play a critical role in the transmission of ARGs, facilitating their spread among bacterial populations and contributing significantly to the challenges faced in infection control (16). The discovery that 98.25% of ARGs subtypes were associated with MGEs underscores the extensive diversity and adaptability of these elements in *E. coli*. This widespread association indicates a robust mechanism for the propagation of resistance, which poses a substantial challenge to current infection control strategies. Moreover, the results that 89 ARGs subtypes were linked to all three types of MGEs, with a particular emphasis on those coding for resistance to aminoglycosides and *β*-lactamases (Supplementary Table S5), underscores the critical need for heightened surveillance and vigilance. These subtypes represent a significant risk due to their ability to spread rapidly across different environments and host species, potentially leading to outbreaks of hard-to-treat infections. The specific association of *IS6* and *Int1* with a broad range of ARGs further highlights the influence of particular MGEs in accelerating the spread of resistance. Notably, the *IS6* family has been identified as crucial in transmitting key resistance mechanisms, including those providing resistance to aminoglycosides and *β*-lactamases. For instance, Wang et al. (44) observed that IS within the *IS6* family were prevalent in MDR bacterial isolates from hospital settings. Their findings indicate that *IS6* not only promotes the spread of resistance genes but also contributes to the genetic diversity of pathogens, thereby enhancing their ability to withstand antimicrobial pressures. Moreover, research by Snaith et al. (45) investigated the dynamics of *IS6* elements in agricultural settings, discovering their critical role in the movement of β-lactamase genes among members of the *E. coil*.

Globalization and international trade have significantly accelerated the dissemination of antibiotic resistant bacterial strains and ARGs across the world (46). This study employed cluster analysis to investigate the global distribution of mobile ARGs, identifying 1,451 OTUs based on 99% homology. Such an analysis offers profound insights into the mobility patterns and impacts of ARGs across various regions. The findings indicated that 4.14% of these mobile ARGs were present in over 20 countries, underscoring their widespread nature. Particularly, resistance genes such as *β*-lactamase *bla*_TEM-163_, and aminoglycosides *aph(3″)-Ib* and *aph(6)-Id* were prevalent in more than 50 countries (Supplementary Table S6), highlighting their potential for global transmission. This widespread distribution is likely influenced by factors such as global trade (47, 48), human migration (49), and environmental dynamics (50). Supporting this hypothesis, further analysis indicated that these globally distributed ARGs primarily originated from human sources, while environmental reservoirs acted as secondary contributors (Figure 6; Supplementary Table S7). Moreover, recent research by Frost et al. (51) also confirmed that global travel and trade significantly contribute to the transnational spread of ARGs, especially in densely populated or highly interconnected regions. Additionally, the analysis of continental abundance variations reveals differing impacts of ARGs across regions. Asia and Europe, characterized by dense populations and substantial antibiotic use, emerged as primary hotspots for ARGs distribution. In contrast, Oceania showed the lowest ARGs abundance, possibly reflecting more rigorous antibiotic stewardship and geographic isolation.

## 5 Conclusion

This study’s comprehensive analysis of extensive *E. coli* genome data has highlighted the prevalent and diverse nature of ARGs within the species, particularly focusing on the widespread distribution of MDR genes and other key categories of ARGs. It revealed significant differences in ARG diversity between high-income and low- to middle-income countries, underscoring the global challenge posed by antibiotic resistance and its correlation with socio-economic factors. Furthermore, the study confirmed the critical role of MGEs in facilitating the spread of ARGs, posing a potential threat to global public health. These findings underscore the urgent need for enhanced surveillance and management of antibiotic resistance on a global scale, as well as the development of new strategies to combat the spread of resistance. Future research should aim to deepen understanding of the mechanisms behind ARG transmission and to develop effective countermeasures across different regions and economic contexts. Moreover, strengthening international cooperation is vital for addressing the challenges posed by antibiotic resistance. Overall, this study provides crucial scientific evidence for formulating global strategies against antibiotic resistance, aiming to foster continuous improvement in global public health.

## Data Availability

Publicly available datasets were analyzed in this study. This data can be found at: the dataset supporting the conclusions of this research is available in the NCBI Reference Se-quence Database, accessible at https://www.ncbi.nlm.nih.gov/refseq/, with the data last retrieved on April 16, 2023.

## Footnotes

^1^ https://www.ncbi.nlm.nih.gov/refseq/

^2^ https://www.rproject.org/

^3^ https://www.omicshare.com

^4^ http://www.cloud.biomicroclass.com/CloudPlatform
