# Supplementary material for: A global perspective on the abundance, diversity and mobility of antibiotic resistance genes in Escherichia coli
Source: Front Vet Sci. 2024 Nov 13;11:1442159. doi: 10.3389/fvets.2024.1442159 (PMC11600533; doi:10.3389/fvets.2024.1442159)
Supplement: Supplementary file 1 [file Data_Sheet_1.docx]

**Supplementary Information**

**A Global Perspective on the Abundance, Diversity and Mobility of Antibiotic Resistance Genes in Escherichia coli**

Yun Qing ^1,2,^^3†^, Zhongai Zou ^4†^, Guolian Jiang ^3^, Lingshi Qin ^3^, Kehui Liu ^1^, Zongbao Liu ^1,2,^^3^*****

^1^ Key Laboratory of Ecology of Rare and Endangered Species and Environmental Protection (Guangxi Normal University), Ministry of Education, Guilin, Guangxi, China

^2^ Guangxi Key Laboratory of Landscape Resources Conservation and Sustainable Utilization in Lijiang River Basin, Guangxi Normal University, Guilin, Guangxi, China

^3^ College of Life Sciences, Guangxi Normal University, Guilin, Guangxi, China

^4^ College of Environment and Public Health, Xiamen Huaxia University, Xiamen, Fujian, China

**† Equal contribution to the study**

*******Corresponding author:**

Dr. Zongbao Liu

Mailing address: College of Life Sciences, Guangxi Normal University, Guilin, Guangxi, China

E-mail address: zongbaoliu@mailbox.gxnu.edu.cn


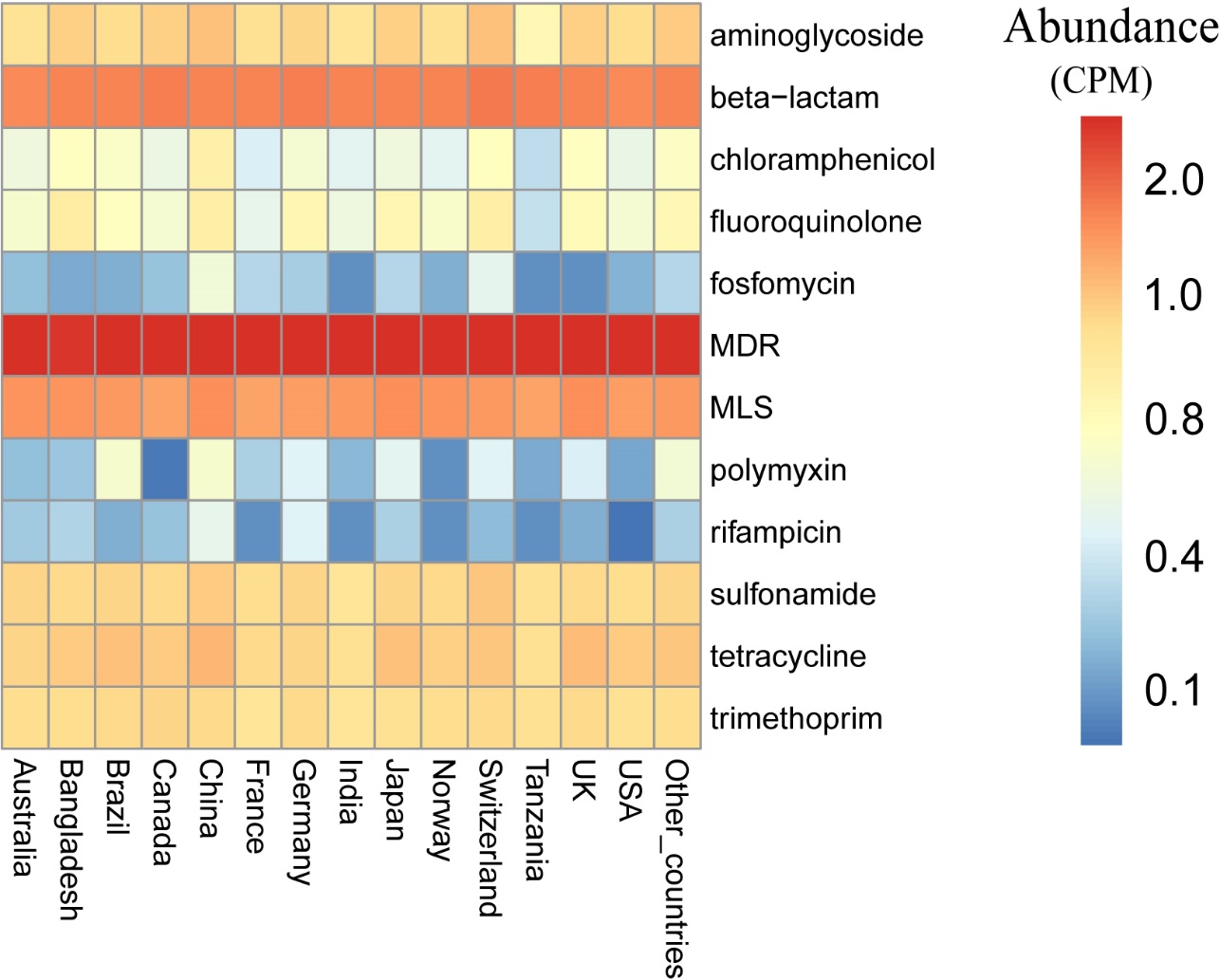


**Figure S1 Abundance of ARG types in E.coil among different countries.** CPM: copies of ARG per million base pairs.


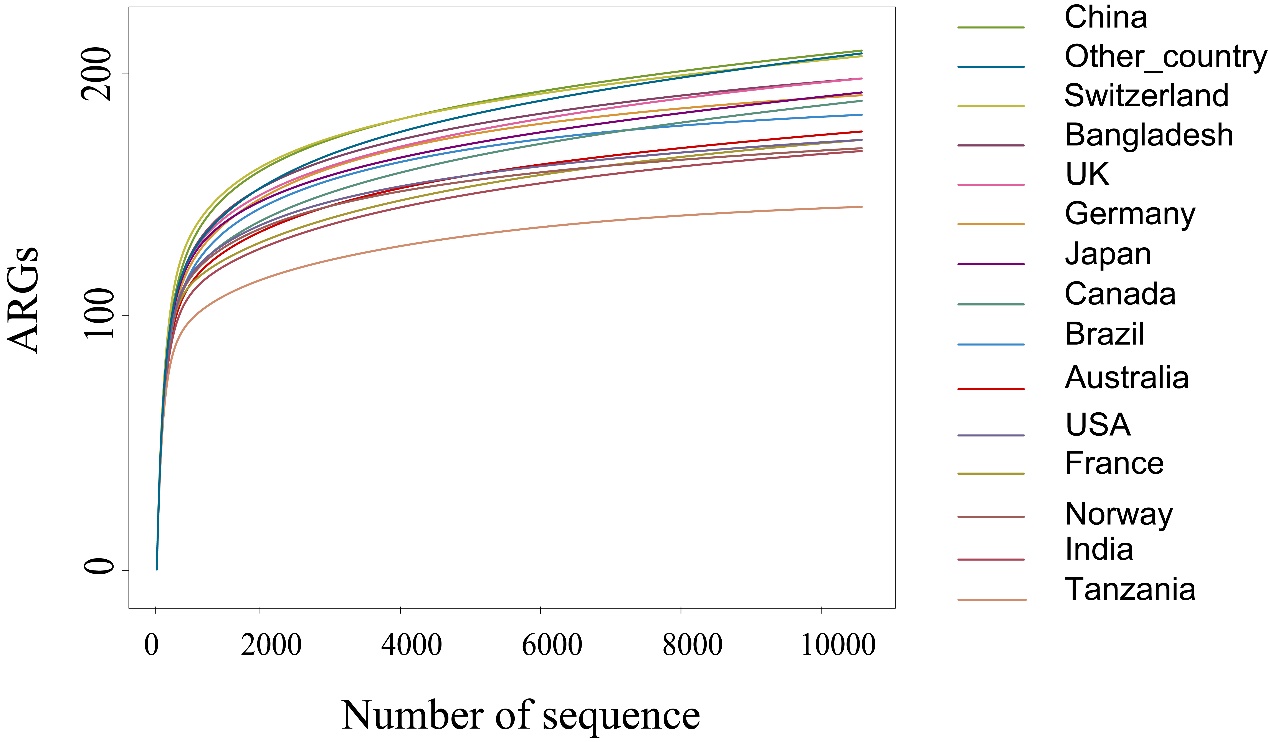


**Figure S2 Rarefaction curve of ARGs across different countries**


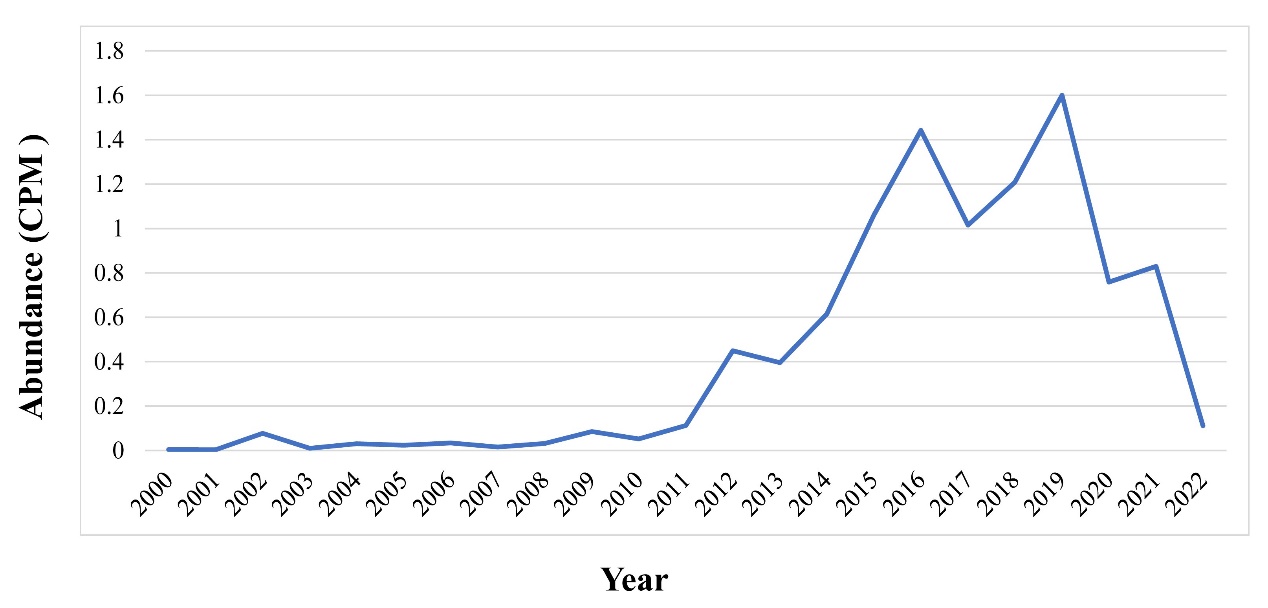


**Figure S3 Temporal trends in ARG abundance in E. coli from China, 2000-2022**


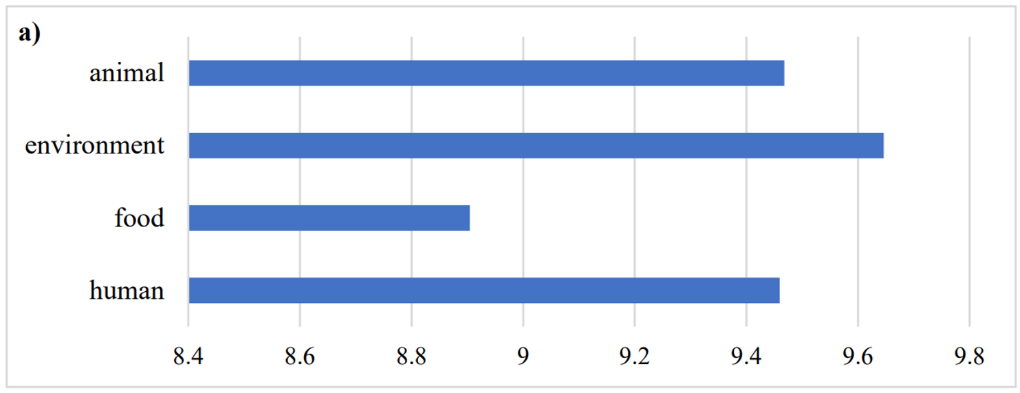


Abundance (CPM)


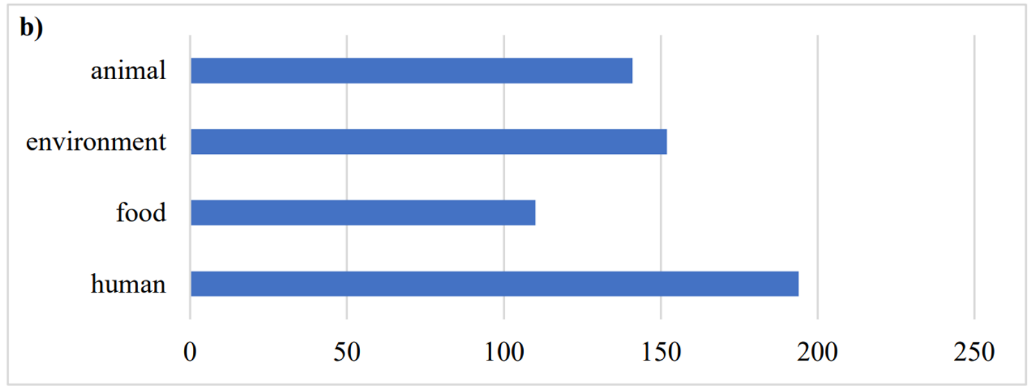


Number of ARG subtype

**Figure S4 Variation in the abundance (a) and diversity (b) of ARGs in E. coil across different sources**


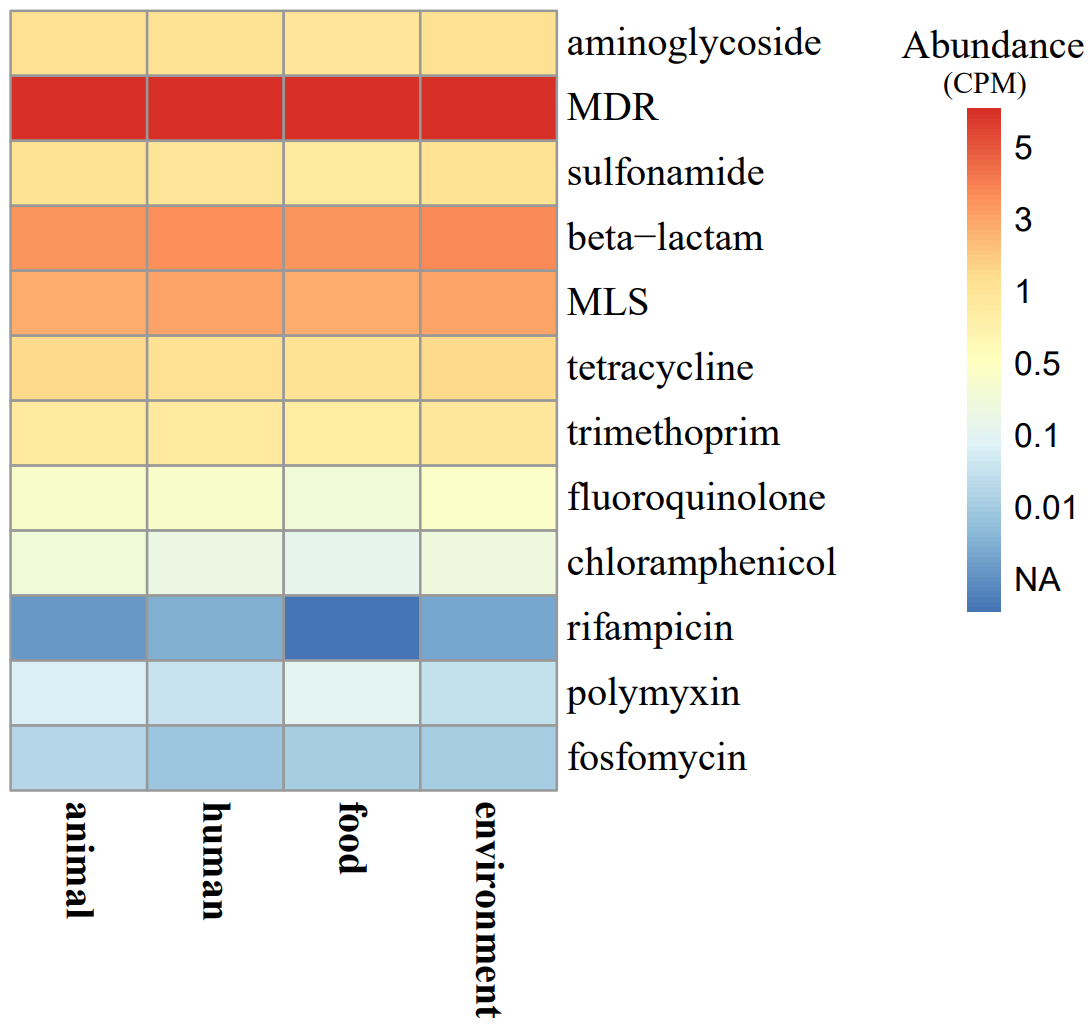


**Figure S5 Abundance of ARG types in E. coil from different sources**


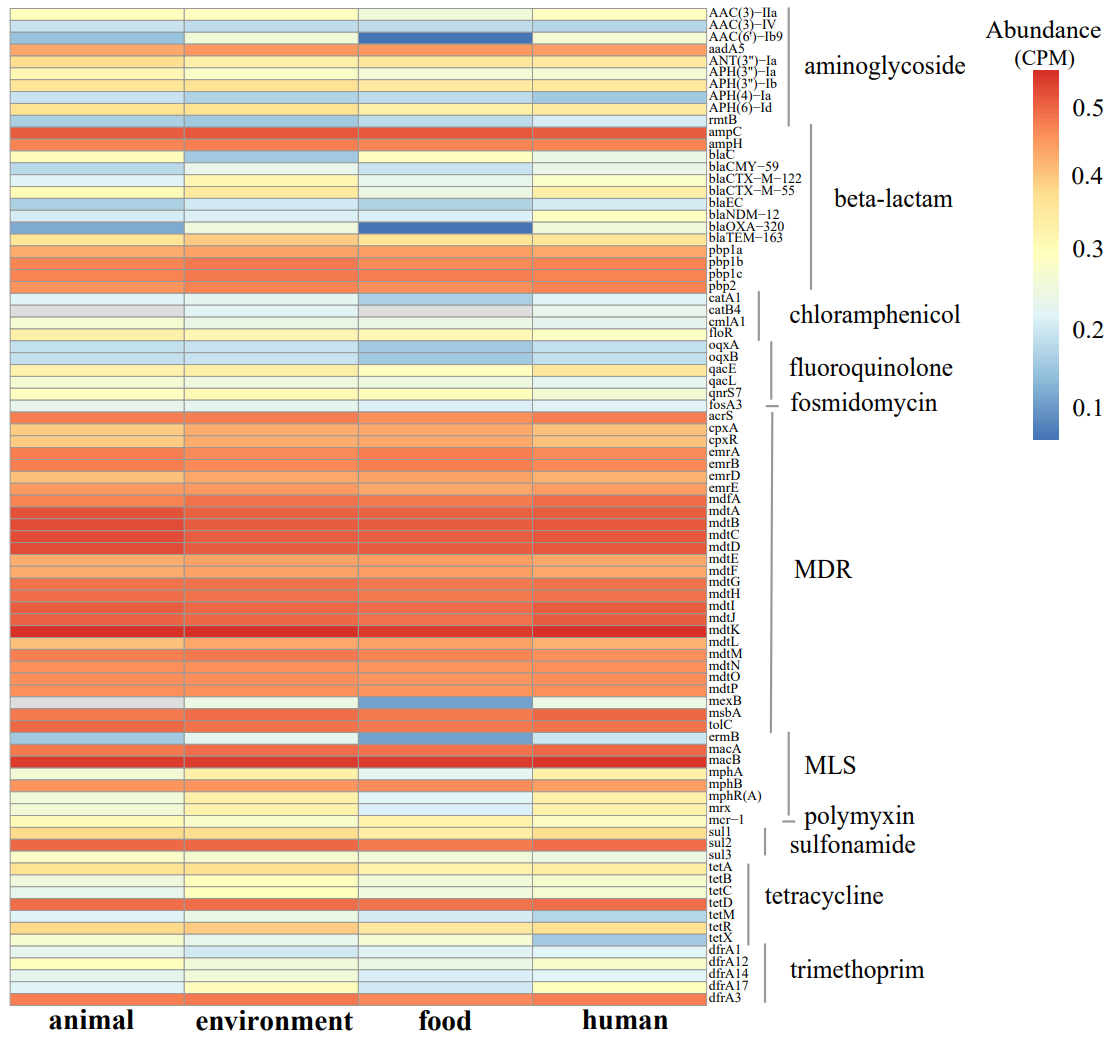


**Figure S6 Abundance of ARG subtypes in E. coil from different sources**


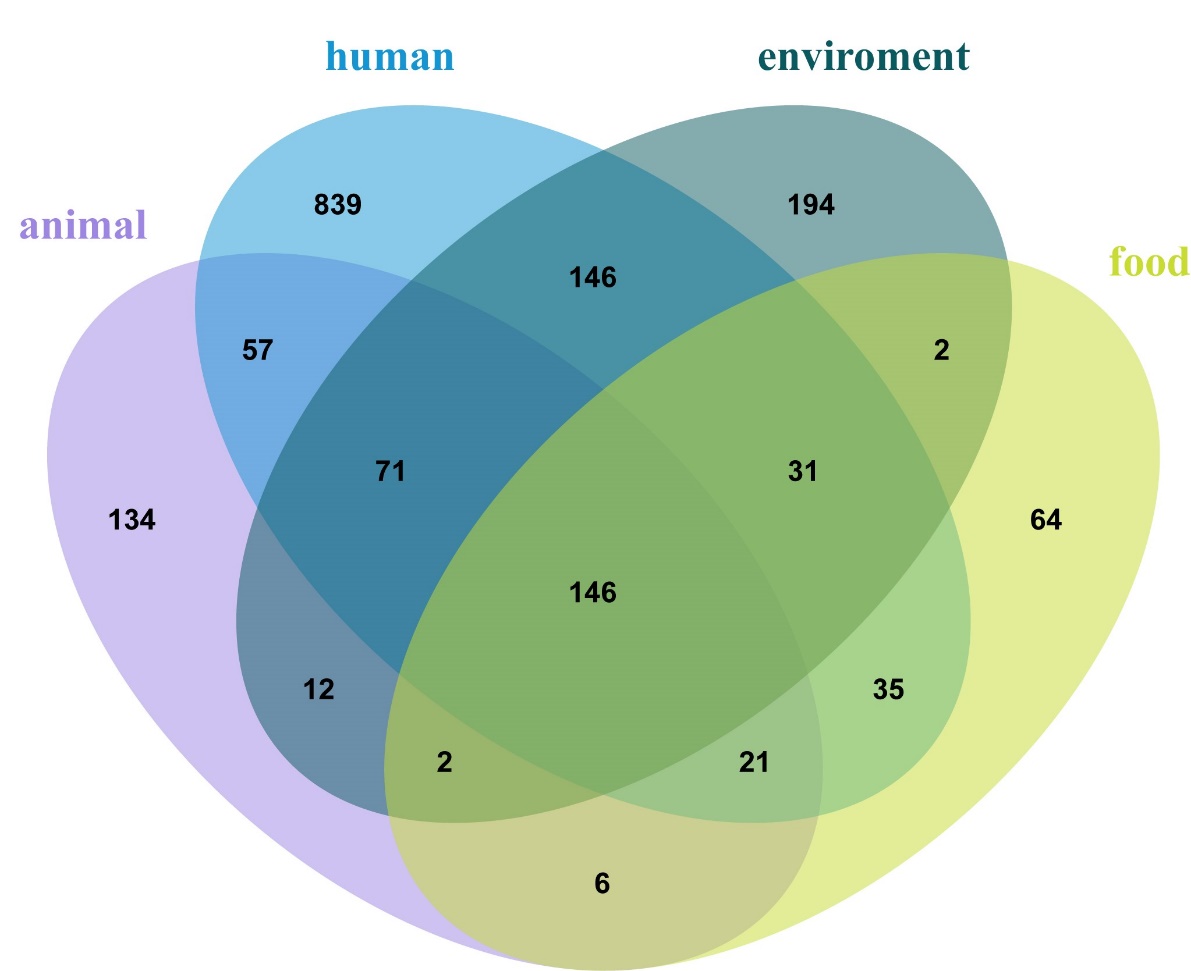


**Figure S7 Venn diagram showing the shared and unique identical or highly homologous ARGs across different sources**
